# Lessons learned from nursing crisis meetings: Qualitative study to evaluate nurses' experiences and needs

**DOI:** 10.1002/nop2.2037

**Published:** 2023-12-15

**Authors:** Sabine Adriana Johanna Josepha op ‘t Hoog, Annemarie Johanna Burgje Maria de Vos

**Affiliations:** ^1^ Academy of Nursing Science and Education Elisabeth‐TweeSteden Hospital Tilburg The Netherlands; ^2^ School of People & Healthcare Studies Fontys University of Applied Sciences Tilburg The Netherlands; ^3^ Centre of Expertise Health, Care & Wellbeing Avans University of Applied Sciences Breda The Netherlands

**Keywords:** COVID‐19, leadership, nursing staff, nursing, organizational structure

## Abstract

**Aims:**

The aim of this study is to evaluate the nurses' experiences with the Nursing Crisis Meetings and to identify nurses' needs regarding the future governance structure.

**Design:**

Qualitative study.

**Methods:**

Two focus groups were conducted in February 2022 with participants of the Nursing Crisis Meetings (*N* = 15). We used thematic analysis to describe themes.

**Results:**

We identified five themes: opportunity to speak up, call for nursing leadership, call for control over practice and autonomy, development of a governance infrastructure and development of the professional nurse role.

**Conclusion:**

Nurses experienced the Nursing Crisis Meetings to be a positive and empowering infrastructure, which facilitates the unique opportunity to speak up and share experiences and concerns. This new infrastructure is a promising strategy to engage nurses during a pandemic and to build on a professional governance structure.

**Impact:**

This paper highlights the need for nurses to speak up and be engaged during the COVID‐19 pandemic and gives a practical example of how to put this infrastructure into practice.

## 1 INTRODUCTION

Nurses take account of almost 50% of the global health care workforce (World Health Organization, 2020). Nurses are critical for a vital health care system and we need to focus more on keeping them on board. This became clear again during the COVID‐19 crisis which has had a major impact on healthcare, especially on nursing practice (Stalpers et al., 2021). During the COVID‐19 crisis, health care organizations set up crisis structures, which had clear top‐down structures. However, to effectively respond to the challenges of a crisis such as a pandemic, nurses should participate and collaborate in multilevel professional governance structures (Hancock et al., 2021; Raso, 2019).

### 1.1 Background

The COVID‐19 pandemic has shone a new light on professional governance as an essential strategy for managing crises (Lal, 2021). Accountability for patient care through engagement of nurses at all levels in practice decisions is a fundamental component of professional governance (Hess Jr et al., 2020). Moreover, early lessons learned during the pandemic showed that strong communication systems are crucial to deal with vital processes, such as the acute care for a new category of severely ill patients (Gómez‐Ochoa et al., 2021; Islam et al., 2020).

During the fourth wave of the COVID‐19 pandemic in 2021, we faced a second major crisis: the shortage of nurses, which underlined the urgency to take care of the well‐being of professionals and make sure they can perform in a healthy environment. Keyko et al. (2016) defines a healthy nurse work environment as a workplace that is safe, empowering and satisfying, and promotes work engagement (Keyko et al., 2016).

Engaged professionals who perform well are able to create their own resources, thereby sustaining a positive gain spiral to further foster work engagement over time (Bakker & Demerouti, 2008). Against the backdrop of an overburdened health care system, work engagement could be a potential avenue to—not only—enhance talent retention but also to create a self‐sustaining resource for employees and ensure high patient care standards. Therefore, organizations should consider leveraging employee engagement, instead of solely focusing on preventing burnout. For example, Goitein (2020) described a clinician‐directed performance improvement program that was designed to give clinicians in a hospital in Santa Fe, New Mexico, protected time and resources to undertake projects that they were interested in, and that could benefit the hospital (Goitein, 2020). The program ultimately resulted in increased clinician morale, engagement and overall hospital quality rating (Wee & Lai, 2022).

To enhance communication and facilitate nurses' input into the Board of Directors' decisions during the crisis, the Nursing Council of a large teaching hospital in The Netherlands initiated a bottom‐up nursing crisis communication structure. Daily Nursing Crisis Meetings facilitated nurses to discuss issues affecting nursing practice, including social distancing, scarce resources, mental health and aggression of visitors (Box 1). Nursing Council members supported nurses in clarifying the problems and empowering ownership and leadership. Specifically, supportive conversations conducted by leaders to review healthcare workers' concerns can help to reduce anxiety and influence system‐wide changes (Adams & Walls, 2020). The Nursing Council acted as intermediary advisor between the nurses and the Crisis Management Team. The objective of this study is to evaluate the nurses' experiences with the daily Nursing Crisis Meetings and to identify nurses ‘needs regarding the future governance structure.

Box 1 **Procedure nursing crisis meetings.**

**Table:** 

Daily meeting from 8:15 to 8:30 AM Each unit is represented by a nurse (max. 15 nurses) A member of the Nursing Council takes the lead during the meeting Standard format of questions: Which problem areas need priority now? What does your team need? What solution do you suggest? Which issues need to be addressed in the Crisis Management Team? The member of the Nursing Council escalates problems and suggestions to the Crisis Management Team

## 2 THE STUDY

### 2.1 Aims

This study aimed to evaluate the nurses' experiences with the Nursing Crisis Meetings and to identify nurses ‘needs regarding the future governance structure.

### 2.2 Design

This qualitative study addresses the following three questions:

1. How do nurses experience the Nursing Crisis Meetings in terms of communication and work engagement?
2. How do the Nursing Crisis Meetings contribute to professional governance?
3. Which lessons learned can be translated into future policy for professional governance structures and processes?

We used a phenomenological approach, because its emphasizes the ‘experiences from the viewpoint of experiencing persons in a life‐world’ (Becker, 1992; Liamputtong & Ezzy, 2005). We conducted two focus groups in February 2022. The findings of this study are reported according to the COnsolidated criteria for REporting Qualitative research (COREQ) (Tong et al., 2007).

### 2.3 Sample/participants

This study took place in a teaching hospital in Tilburg, The Netherlands (annually 58,467 admissions and 166,399 outpatient visits; 1550 nurses; 394 doctors). We used a convenience sample of nurses who attended the Nursing Crisis Meetings between 22 November 2021 and 22 January 2022. Due to the context of the crisis situation, we expected a low response. Therefore, we decided to use a convenience sample for our study (Boeije & Bleijenbergh, 2014; Braun & Clarke, 2006). By using the attendance list of the Nursing Crisis Meetings, we were able to approach the potential respondents by email and invite them to participate in the study. Inclusion criterion was attendance of the Nursing Crisis Meeting at least once.

### 2.4 Data collection

Two hybrid focus groups were conducted to explore the nurses' experiences with the Nursing Crisis Meetings (Lobe & Morgan, 2021). Both focus groups were held in Dutch. Data were collected face to face or using a video link using Microsoft Teams owing to pandemic restrictions. An experienced, trained and independent unbiased moderator led the conversations to the study areas by using an interview guide (Appendix A). Both researchers (SAJJoH and AJBMdV) were present during the focus groups to observe non‐verbal communication. The participants were encouraged to express their views and opinions freely. The time for the focus groups ranged from 40 to 60 min. The focus groups were audiotaped with the participants' permission and transcribed verbatim and anonymously (Table A1).

### 2.5 Ethical considerations

Participants received a digital information letter about the study and were assured confidentiality and their right to withdraw, without any consequences. Written informed consent was obtained from each participant prior to the focus group commencement. Data were processed anonymously by using pseudo‐anonymized study numbers. Data are stored in the hospital's secure digital data management system called Research Manager. The Medical Research Ethics Committees REDACTED (REDACTED) and the Hospital Board of Directors (REDACTED) granted approval for the study.

### 2.6 Data analysis

We performed an inductive qualitative thematic analysis using the six‐phase guideline of Braun and Clarke (2006). First, transcripts were read line‐by‐line and open codes and labels were assigned (phase 1 and 2). Secondly, we grouped the codes into categories using axial coding (phase 3). Data were analysed in an iterative way by rereading and coding data and sorting these into categories. Two researchers experienced in qualitative studies (SAJJoH and AJBMdV) independently analysed both transcripts and discussed the differences until consensus was reached. The categories were reduced to an abstract level of themes using a visual analysis scheme (phase 4 and 5). To maintain integrity and trustworthiness, the researchers discussed until they reached a consensus on the final main themes to finally report on the themes (phase 6).

We used ATLAS.ti version 9.0.0.214 (ATLAS.ti 9 Windows ‐ User Manual (atlasti.com) as a data management tool to perform the analyses and visualize the resulting themes.

### 2.7 Rigour

To maximize the credibility of the study, we conducted a member check to ensure that the results accurately reflected the respondents' lived experiences. After phase 6 we sent the findings per mail to all participants and asked for their reflection (Birt et al., 2016).

## 3 FINDINGS

Fifteen respondents participated in the two focus groups. Focus group 1 was held on 3 February 2022 with 10 participants. Focus group 2 was held on 8 February 2022 with five participants (seven respondents signed up, but two cancelled due to COVID‐19 contamination). All respondents were Registered Nurses and worked during the study period as a nurse in the units. Five respondents worked in a COVID‐19 unit. Two respondents worked in the Intensive Care Unit, and two respondents worked in the Emergency Room. The other six respondents worked in general units.

**FIGURE 1:**
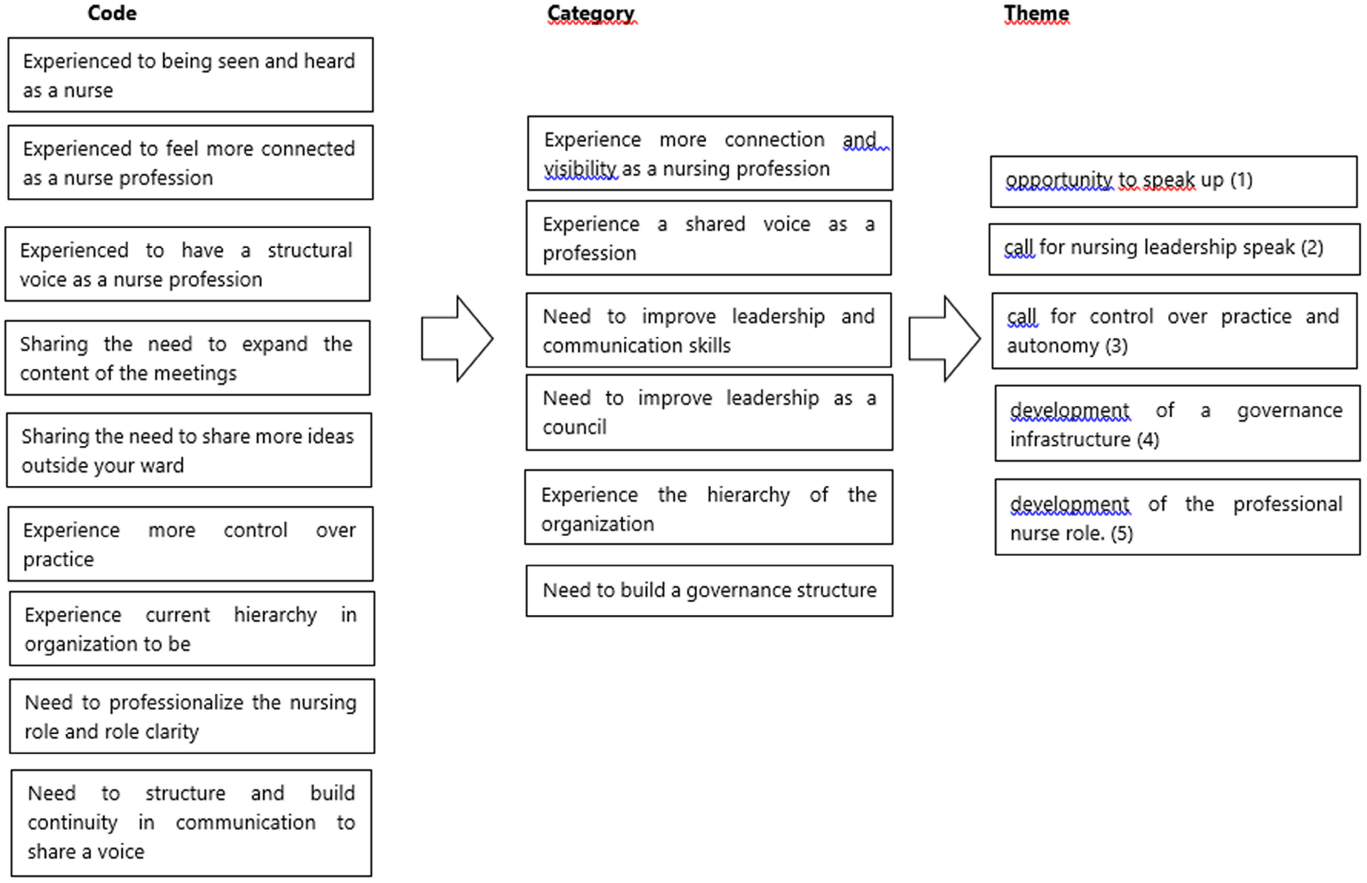
Code‐tree of the five themes, categories and codes.

We identified five themes: opportunity to speak up, call for nursing leadership, call for control over practice and autonomy, development of a governance infrastructure and development of the professional nurse role. We describe the findings with additional participants' quotations to illustrate the themes and to highlight the consistency between the data. The code‐tree is visualized in Figure 1.

### 3.1 Opportunity to speak up

Respondents mentioned both positive and negative experiences regarding their participation in the Nursing Crisis Meetings. A key theme that emerged was to be heard. To be heard by colleague nurses and by the Board of Directors. In addition, nurses felt that they had the opportunity to speak up about their concerns and daily work experiences at the Nursing Crisis Meetings. They shared their experiences mostly about their work in their unit, about patient care and the struggle with the high patient flow. Sharing experiences led to mutual understanding and empathy.

‘I think that in the big picture it is really very valuable to be able to share experiences, to gain insight into how things work in other units in the broadest sense of the word, right? Normally you look for the connection in your own work environment. Also in terms of work pressure and things like that, I think the Nursing Crisis Meetings really did contribute to create a connection between nurses from different units. Nurses from surgery units helped nurses of other units and vice versa, when they shared their experiences of how to deal with high work pressure’. (respondent 13).

Moreover, nurses encouraged each other to share lessons learned and new initiatives. For example, nurses shared their lessons learned with creating and implementing a buddy system, which meant that nurses teamed up—as buddies—to look after each other during a shift. Another example is the use of the three‐questions‐method to cope with the emotional burden during COVID‐19 (De Bot & De Vos, 2021) This method implies that nurses ask each other three questions: (1) What today's events do you remember? (2) How do you feel (physically and mentally)? (3) Do you have enough support? Both initiatives were adopted in different units. Sharing lessons learned and new initiatives contributed to moments of reflection and created connection between nurses from different units.

‘It really gets a conversation starting, and I think that's a good thing and especially in times of crisis. But I also think ‐ in everyday work ‐ you get the opportunity to speak up and being heard’. (respondent 7).

In addition, nurses mentioned the concept of experiencing more connection as a profession. As a result of the Nursing Crisis Meetings, they felt more connected with each other on themes they all struggled with, which empowered them to act as one professional group rather than as an individual nurse or team. This feeling of being part of a professional group added to a sense of togetherness, which contributed to collaboratively having impact on addressing practical problems and challenges.

When you do it together … it does feel like you have an impact ‐ … Well it is a different perception, a different experience’. (respondent 2).

If you have ten nurses all saying the same thing, that just makes it that much stronger and then you do notice during the Nursing Crisis Meeting. You all struggle with the same thing. If you just … stand for it together, that's something that's much stronger than when you're just trying to solve it as an individual team. (respondent 2).

One respondent mentioned that sharing experiences and solutions had a positive impact on their resilience capacity.

That sometimes in the morning I thought, how are we going to get the job done today as a team? And then during the Nursing Crisis Meeting I heard similar experiences in other units. And sometimes help from other units arrived, because they had heard during the meeting how busy we were. That makes you think, well, I've got some air… (respondent 2).

### 3.2 Call for nursing leadership

Respondents experienced nursing leadership through sharing lessons learned and reflecting on experiences during the Nursing Crisis Meetings. In addition, they critically questioned each other and provided constructive feedback, which inspired not only the attending nurses but also the whole team through the feedback loop. However, the lack of guidance was also mentioned, as some nurses experienced the meetings as a ‘moaning moment’. Two respondents expressed the need for a strong chairperson during the Nursing Crisis Meetings to facilitate an effective meeting.

I think that as nurses, we definitely need to show more leadership and put ourselves more in in front. But, I think that it also works both ways and that you also need to be facilitated in terms of time and support. (respondent 13).

### 3.3 Call for control over practice and autonomy

Ten respondents indicated that nurses became increasingly aware of the organizational structure when they noticed the Nursing Council escalating issues to the Crisis Management Team with the request to address these. As a result, nurses gained more understanding and increasingly showed appreciation towards the Board of Directors. More importantly, nurses experienced increased control over their own practice by seizing the opportunity to speak up and share their concerns.

By giving information during the Nursing Crisis Meeting, some problems were immediately reported to the Board of Directors and then it was solved in the afternoon. And then you think, so it is possible in this organization. If you work with short lines, the problems remain current. Given our drive not to settle for anything less, we stayed on top of things. I think that's where the power lies. (respondent 10).

This is how it really is in daily practice. How are we doing as nurses? Which struggles do we face? What do we think is important? Get these questions being answered, that's the difference. (respondent 8).

The Nursing Crisis Meetings were seen as an alternative route for putting topics on the agenda and address their experienced problems of daily practice. Normally, a team leader would gather these topics and share them with managers. However, with the implementation of the Nursing Crisis Meetings, nurses were able to deliver their input, at the same time suggest and share interventions, which they considered effective. This ‘novel process’ contributed to a sense of autonomy among nurses. The respondents expressed their appreciation about this ‘short communication line’. However, they also indicated that the Nursing Council should be more proactive in sharing the final decisions, because they experienced a lack of feedback regarding the result of their input.

There is just one neutral person giving input to, for example, the team leader who often looks at things from a different perspective and who has other interests in things and I had the idea here that yes, the Nursing Council is on our side. (respondent 7).

I think, as nurses that we should have a voice anyway, always. (respondent 2).

### 3.4 Development of a governance infrastructure

In addition to experiences, the respondents expressed the need to continue the Nursing Crisis Meetings after the crisis. They expressed the need to continue sharing experiences among the nursing staff and to maintain having a voice, have a say in their daily work.

The Nursing Council rapidly implemented the Nursing Crisis Meetings to gather input from nurses during the COVID‐19 crisis. The respondents unanimously indicated the need for continuing the Nursing Crisis Meetings, in a different format. They suggested redesigning the structure of the meetings to facilitate sharing experiences, exploring new themes and remaining involved in future decision‐making processes. Connection to the Nursing Council was important according to the respondents, because the Council had an advisory role towards the Crisis Management Team. They indicated that the Nursing Council should increase their visibility by continuing the current meetings (or in a new format), which they consider to be an effective strategy for improving nursing leadership and control over practice.

### 3.5 Development of a professional nurse role

The respondents expressed the need for clarifying the nursing role and their participation in the Nursing Crisis Meetings. Clarity of the members' role is needed to develop deeper connection, leadership and ownership when implementing the Nursing Crisis Meetings as an infrastructure for actual control over practice. Nurses indicated that the Nursing Council and the nurses should collaborate, discuss and define their roles, for example about retrieving information from colleagues. For some nurses, it was not clear that by attending the Nursing Crisis Meetings, they were assigned tasks and responsibilities.

In what role did you participate in the Nursing Crisis Meetings? As an individual or as a representative of your team? That makes a difference. (respondent 2).

When I think of the Nursing Council, communication is everything, so I think. (respondent 4).

Several respondents expressed that they expected more leadership from nurses who attended the Nursing Crisis Meetings. Overall, respondents mentioned the need for more leadership in general, as a profession. On the other hand, there was an expectation towards each other in the Nursing Crisis Meetings. If a nurse was ‘complaining’ during a meeting, respondents felt that she was not suitable to participate, because she distorted the common interest.

Sometimes I thought it was really a moment for complaining…I wouldn't say that complaining is the goal for these meetings. Fifteen minutes are very precious for nurses. (respondent 6).

## 4 DISCUSSION

This study explores nurses' experiences with the Nursing Crisis Meetings and their needs regarding the future governance structure. The Nursing Crisis Meetings gave nurses the opportunity to speak up and share their concerns, which they appreciated and perceived to be a positive development both in the crisis structure and the organization as a whole. In a recent editorial Jackson (2022) holds a strong plea for nurses to speak up. It should be ‘business as usual’ she proclaims (Jackson). Although it requires confidence and courage, speaking up reflects exactly what society needs from nurses: critical thinkers, change agents and problem solvers, who stand up and speak out right from the bedside to even broader more societal‐level advocacy (Jackson).

The implemented Nursing Crisis Meeting was a new communication structure for the nurses in this hospital. Before the COVID‐19 pandemic, nurses from different units did not structurally discuss issues or exchange experiences. Several studies describe the ‘organizational deafness’ during the COVID‐19 pandemic, which refers to the organizations' ignorance of their nurses' concerns (Adams et al., 2020; Maben et al., 2022; Mizumoto et al., 2022; Wynter et al., 2022).This has been a feature of nurses' workplaces for decades (Rasmussen et al., 2022). The new infrastructure was implemented a year after the COVID‐19 outbreak in response to the ‘organizational deafness’. Nurses experienced that the Nursing Crisis Meetings were valuable and empowering to speak up and share concerns. Given their positive experiences, they indicated that this structure should be imbedded in the organizational structure. An important argument is that nurses experienced higher level of engagement and felt more in control of their daily practice. Nurses ‘input was quickly and effectively acted upon by the Crisis Management Team including members of the Board of Directors. In times of crisis, including pandemics and nursing shortages, the call to the Board of Directors is to prioritize the value of professional governance to ensure nurses’ input into the crisis management. In addition, professional governance validates and contributes to the engagement of nurses in decision making (Porter‐O'Grady & Pappas, 2022). A recent review reported statistically significant positive outcomes in employees' ability to participate in decision making and experienced sense of control over practice after implementing new councils on both organization and unit levels (Kanninen et al., 2021).

Our results indicate that nurses increasingly call for control over practice. Control over nursing practice is a democratic process facilitated by a visible, organized and supportive structure. The structure provides nurses input and involvement in decision making concerning clinical policies and problems and personnel issues, which have an effect on nurses. Control over nursing practice, for instance in the form of a nurse practice council, will only lead to the desired outcomes if nurses have the authority to take control over their daily practice (de Brouwer, 2019; Laschinger & Finegan, 2005; Laschinger & Wong, 1999). Several studies claim that autonomy affects the innovative behaviour of nurses (Knol & Van Linge, 2009; Renkema et al., 2021). To move forward in a (post‐) pandemic era, facing nurses shortages and rebalance the workforce, nurses need to be engaged in all decisions regarding the future nursing practice (Feistritzer et al., 2022).

## 5 STRENGTHS AND LIMITATIONS

This study has, like all qualitative case studies, limitations that need to be acknowledged and addressed. First, the findings of this study represent the experiences of a purposeful sampled group of respondents of one Dutch hospital. The analysis of the focus group data provided us insight in nurses' experiences with professional governance in a crisis. Yet, generalizing our findings must be done with caution. Involving a larger group of respondents from different hospitals (in diverse countries), would give more understanding. Second, the experiences of our respondents regarding professional governance might have been affected by nursing shortages and high levels of stress, anxiety, physical exhaustion, due to the nature of the global pandemic. Third, due to restrictions of the COVID‐19 pandemic we conducted hybrid focus groups, which may have affected the smoothness of the interactions. In addition, we may have missed important non‐verbal communication from the online respondents (Mann & Lundrigan, 2022; Tremblay et al., 2022).

To lead the nursing profession forward, nurses need to recognize the current transition from being managed as a hospital based, subordinated employee group, towards a profession driven by effective strategies as professionals and leaders. Building a bottom‐up communication structure is one of the first steps towards a professional governance structure aimed at strengthening the nursing profession in the post‐pandemic era. A Nursing Council has the unique position to act as intermediary advisor between the nurses and the Board of Directors and therefore has the responsibility to enable nurses to speak up and make it ‘business as usual’.

## 6 CONCLUSION

Nurses experienced the Nursing Crisis Meetings to be a positive and empowering infrastructure to speak up and share their experiences and concerns. This new infrastructure is a promising strategy to engage nurses during a pandemic. Further research should give insight into the effectiveness of this infrastructure in terms of nursing leadership, control over practice and autonomy, and development of the professional nurse role.

## AUTHOR CONTRIBUTIONS

Sabine Adriana Johanna Josepha Op ‘t Hoog, corresponding author; Conceptualization, Methodology, Software, Validation, Formal analysis, Investigation, Data curation, Writing—Original Draft, Visualization, Project administration, Funding acquisition. Annemarie Johanna Burgje Maria de Vos; Conceptualization, Methodology, Software, Validation, Formal analysis, Investigation, Data Curation, Writing—Original Draft, Writing—Review and Editing, Visualization.

## FUNDING INFORMATION

This research did not receive any specific grant from funding agencies in the public, commercial or not‐for‐profit sectors.

## CONFLICT OF INTEREST STATEMENT

The authors declare that they have no conflicts of interest.

## 7 ETHICS STATEMENT

The local ethics committee (Medical Research Ethics Committees of Noord‐Brabant, NW2022‐06).

## NO PATIENT OR PUBLIC CONTRIBUTION

No patients, service users, caregivers or members of the public were involved in this study.

## Data Availability

The data that support the findings of this study are available on request from the corresponding author. The data are not publicly available due to privacy or ethical restrictions.

## APPENDIX A

### A.1

**Interview guide: The lessons learned on a Nursing Crisis meeting. A qualitative study to evaluate nurses ‘experiences and needs.**

Aim of research

At the beginning of the fourth wave of the COVID‐19 pandemic, we are facing a second major crisis in addition to the COVID‐19 pandemic: the shortage of nurses. To put lessons learned into practice, the Nursing Council (Dutch, Verpleegkundige Advies Raad) launched a bottom‐up nurse crisis communication structure to encourage the engagement of nurses in the crisis structure of the hospital. Members of the nursing council supported nurses in clarifying issues and strengthening ownership and leadership and acted as an intermediary between nurses and crisis management team. The purpose of this study is to evaluate nurses' experiences with the nursing council and to identify nurses' needs regarding the future consultation structure.

**Skills and competences moderator focusgroup interview (Doody et al.,** **2013** **).**

**Skills**

‐ Creating an open and auction atmosphere.

‐ Create moments of silence to encourage participants to formulate experiences, explore opinions, draw conclusions.

‐ Focus on the main topic, keeping an overview of the discussion.

‐ Keeping all participants involved in the discussion.

‐ Communication skills: explore, question, listen, summarize, restate, refine, clarify.

‐ Switching between phases and levels of discussion.

‐ Time management: dividing time between topics, distributing time among participants, monitoring time, introducing purpose of focus group quickly and efficiently.

**Attitude**

‐ Objective a neutral, not giving advice or opinions on the subject.

‐ Open: inviting and curious, respectful of different opinions and points of view, non‐judgmental.

‐ Attentive to (the importance of) dialogue among participants.

‐ Clear about the responsibilities of the moderator and participants.

‐ Enthusiastic.

‐ Leading, but not dominant.

‐ Focused on the group process.

‐ Open to feedback on own performance.

**TABLE A1:** Topic list (Raat et al., 2019).

Introduce the conversation with the information and questions below. Follow the order indicated.	
Introduction	Thank you for participating in this study and take the time to do so. You should have received an information letter. Is that correct? Are there any questions about the information of this focusgroup?
**Informed Consent**	**Sign and return forms** Offering pens and forms
Introducing the topics	During this focusgroup we ask you about your experiences with the nursing crisis meetings you did participate.
Explain to participants the aim of the study	Several focus groups will be held with nurses working in this hospital who participated in the nursing crisis meetings. The aim is to explore the nurses ‘experiences of this communication strategy and needs to future strategies to engage nurses. Although participants individually answer the facilitator's questions, they are encouraged to talk and interact with each other.
Explain to participants data will be processed anonymously and confidentially	By processing the data, your name will not be mentioned. That way, no one will be able to find out later in the research report exactly what you said. The things you tell us will only be used for this project and therefore cannot be traced back to individuals.
When a participants wants to quit	If you decide during the focus group that you do not want to continue, you can say so, any time. Also after this focus group participants are free to withdraw by from the study at any time.
Time schedule	When all topics have been discussed and no more information is shared the conversation end earlier.
The conversation will be audio taped	The conversations during this focus group will be audio taped. This ensures that we do not have to write down much now but can listen to you. In fact, our conversation will be transcribed later. It is required by law that we must ask your permission if we are using audio recording. Therefore, I will ask you in a moment, when the recorder is running, if you give permission for this conversation to be recorded.
	**Start audio recording** Do you give permission for audio tape this conversation?

Introducing the questions	
General introduction	Can you tell something about your work as a nurse during the COVID‐19 outbreak in this hospital? ‐ Which department do you work at? ‐ Can you explain the context?
Experiences	Looking back at the times when you participated in the nursing crisis meeting, − ‐ can you share your experiences on these? ‐ What did you think of these meetings?
Experiences	Which benefits did you and your team experience because of these nursing crisis meetings?
Experiences	What did you experience as positive? Which improvements would you suggest?
Experiences	Did these nursing crisis meetings changed anything for you as a nurse? Can you explain what and why, please explain?
Experiences	Did the existence of these nursing crisis meetings accomplished anything on your ward in patient care? If so, what and how, please explain?
Needs	How do you think these nursing crisis meetings should continue after the crisis structure?
Needs	What should be the aim? What conditions would you prefer?
Possible connectors to the main question	What did you think of how this was done? That's interesting, can you talk a little bit more about that? Non‐verbal encouragement of storytelling.
Look for clues in the story to ask further questions about what the respondent is saying.	Auxiliary questions: You mention (….), can you explain that? Can you give an example of a situation where that played out? You just said (….). Can you clarify that? What happened then? You just said (…). What do you mean by that? What are your experiences with that? What kind of feeling gave this to you and why? How do you think about that? What do you think about that? Did I understand correctly that….?
Closing the conversation	Is there anything else you would like to add to this conversation? Are there any things that were not covered in the conversation that you think are important to mention? How do you look back on this conversation in this focus group?
Further process	This conversation during this focus group will be typed out verbatim. These key points will be compared with the key points we extract from the other focus groups.
Thank you	I would like to thank you for your time and participation in this focus group. I hope you feel that you were able to tell your story and that I listened carefully to it.
Contact	For any questions you can reach out for one of the researchers: Sabine op ‘t Hoog or Annemarie de Vos
